# Macrophage and adipocyte interaction as a source of inflammation in kidney disease

**DOI:** 10.1038/s41598-021-82685-4

**Published:** 2021-02-03

**Authors:** Cristina Martos-Rus, Goni Katz-Greenberg, Zhao Lin, Eurico Serrano, Diana Whitaker-Menezes, Marina Domingo-Vidal, Megan Roche, Kavitha Ramaswamy, Douglas C. Hooper, Bonita Falkner, Maria P. Martinez Cantarin

**Affiliations:** 1 Division of Nephrology, Department of Medicine, Sidney Kimmel Medical College at Thomas Jefferson University, 833 Chestnut Street, Suite 700, Philadelphia, PA 19107 USA; 2 Sidney Kimmel Cancer Center, Thomas Jefferson University, Philadelphia, USA; 3 Cancer Biology and Neurological Surgery, Thomas Jefferson University, Philadelphia, USA

**Keywords:** Cell biology, Immunology

## Abstract

In obesity, adipose tissue derived inflammation is associated with unfavorable metabolic consequences. Uremic inflammation is prevalent and contributes to detrimental outcomes. However, the contribution of adipose tissue inflammation in uremia has not been characterized. We studied the contribution of adipose tissue to uremic inflammation in-vitro, in-vivo and in human samples. Exposure to uremic serum resulted in activation of inflammatory pathways including NFκB and HIF1, upregulation of inflammatory cytokines/chemokines and catabolism with lipolysis, and lactate production. Also, co-culture of adipocytes with macrophages primed by uremic serum resulted in higher inflammatory cytokine expression than adipocytes exposed only to uremic serum. Adipose tissue of end stage renal disease subjects revealed increased macrophage infiltration compared to controls after BMI stratification. Similarly, mice with kidney disease recapitulated the inflammatory state observed in uremic patients and additionally demonstrated increased peripheral monocytes and inflammatory polarization of adipose tissue macrophages (ATMS). In contrast, adipose tissue in uremic IL-6 knock out mice showed reduced ATMS density compared to uremic wild-type controls. Differences in ATMS density highlight the necessary role of IL-6 in macrophage infiltration in uremia. Uremia promotes changes in adipocytes and macrophages enhancing production of inflammatory cytokines. We demonstrate an interaction between uremic activated macrophages and adipose tissue that augments inflammation in uremia**.**

## Introduction

Patients with end stage renal disease (ESRD) have high circulating levels of inflammatory markers such as CRP, IL-6, TNFα, and IL-1α^1,2^. Clinical evidence of increased inflammation is associated with poor outcomes in patients with ESRD on hemodialysis, peritoneal dialysis, and in chronic kidney disease (CKD) after transplantation^3–8^. Inflammation plays a role in the development of multiple complications of kidney disease including protein energy wasting^9^, anorexia^10^, anemia, vascular calcification^11^, and endocrine disorders including insulin resistance. Inflammation is also now considered a novel and independent risk factor for cardiovascular disease^12,13^.

The process by which kidney disease causes inflammation is poorly understood and many potential mechanisms have been described. We previously demonstrated greater macrophage infiltration in adipose tissue of uremic patients compared to normal kidney function controls^14^. In obesity, adipose tissue macrophages (ATMS) are the most common white blood cells that infiltrate adipose tissue. The number of macrophages present in obese adipose tissue directly correlates with degree of adiposity and with adipocyte size in both human subjects and mice, with no differences present between subcutaneous and visceral adipose depots. Macrophages in adipose tissue are a significant source of circulating TNFα and IL-6. In humans with non-inflammatory conditions, adipose tissue contributes up to 35% of the total circulating IL-6 levels^15^. In inflammatory conditions such as obesity, the production of inflammatory cytokines by the adipose tissue can increase up to 50% of the circulating pool^16^. Therefore, it is plausible that the contribution of adipose tissue inflammatory cytokines to the overall inflammatory states seen in uremia is significant. A close relationship between macrophages and adipocytes has been demonstrated in obesity. Hypertrophic/hyperplastic adipocytes produce chemokines that will promote macrophage infiltration in obesity^17^. Once macrophages migrate to the obese adipose tissue, they acquire an inflammatory phenotype^18^. Inflammatory ATMS in obesity are the main source of adipose tissue inflammatory cytokines, which promote detrimental metabolic effects associated with obesity^19^ including insulin resistance and adipocyte dysfunction^16^. However, adipose tissue dysfunction in uremia and its potential contribution to inflammation has not been characterized. Furthermore, the role of adiposity in ESRD and its contribution to patient outcomes has been a long-standing debate. Traditionally, obese ESRD patients on dialysis were thought to have a survival advantage^20,21^, but this view has been recently challenged. Stenvinkel et al. reported evidence that inflammation modifies the relationship of obesity with mortality in ESRD patients^22^, supporting the concept that inflammation in ESRD patients is associated with excess morbidity independent of adiposity. With this background, we designed a series of experiments to investigate macrophage and adipose tissue inflammatory responses to uremia. Our experiments include in-vitro cell models with exposure to uremic serum, an in-vivo mouse model of uremia, and human tissue studies.

## Results

### Adipokine expression and glycolysis in 3T3-L1 adipocytes is increased after uremia exposure

**Figure 1:**
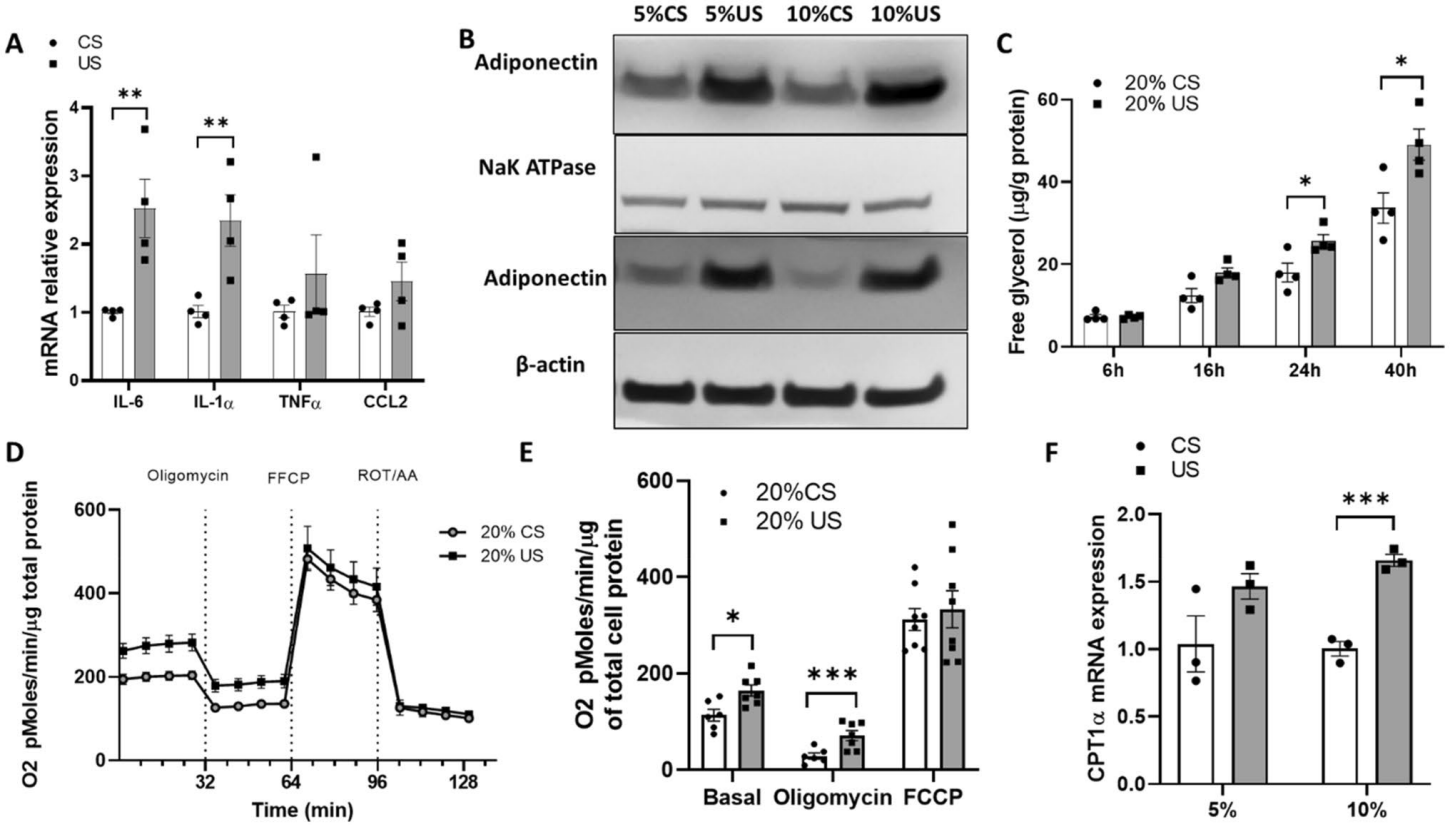
Adipokine expression and glycerol release in 3T3-L1 adipocytes is increased after uremic serum exposure. Differentiated 3T3-L1 cells were exposed to different concentrations of control and uremic serum. (**A**) IL-6, IL-1⍺, TNF⍺ and CCL2 mRNA expression levels assessed by RT-qPCR after cells were exposed to 10% uremic and 10% control serum. (**B**) Adiponectin content assessed by western blot in membrane and cytosolic compartment after 3T3-L1 cells were exposed to control and uremic serum (cropped membrane, full length blots are presented in Figure S4). (**C**) Free glycerol in media from 3T3L1 after treatment with control and uremic serum for 24 h. (**D**,**E**) OCR measurements in 3T3-L1 cells treated with control and uremic serum for 24 h. (**E**) Shows the representative time course data for OCR. (**E**) Shows the aggregated data. Data from OCR experiments represent the mean of four technical replicates from two representative experiments. (**F**) CPT-1α mRNA expression levels assessed by RT-qPCR after cells were exposed to 5–10% uremic and control serum. Unpaired t test, n = 4 for (**A**–**D**), experiments were done in triplicate, error bars represent SEM. *p < 0.05 **p < 0.01 C, control. U, uremic. Full length blots are presented in Figure S4.

To determine the effects of uremia on adipose tissue function, we studied adipokine production in a 3T3-L1 cell model. Cells exposed to uremic serum increased expression of *IL-6, IL-1α,* and adiponectin (Fig. 1A). Since uremic patients have increased circulating adiponectin levels^1^, we studied adiponectin expression in cell membrane and cytosolic fractions to delineate the origin of the higher adiponectin levels observed in 3T3-L1 cells exposed to uremia. As seen in Fig. 1B, both the membrane and cytosolic fractions of 3T3-L1 cells exposed to uremia demonstrated higher adiponectin protein reflecting increased adiponectin production as well as adiponectin binding to the cell membrane. Since inflammation can promote glycolysis and patients with advanced kidney disease have higher circulating free fatty acids, we studied adipose tissue catabolism, specifically lipolysis levels. Figure 1C shows increased glycerol release in 3T3-L1 cells exposed to uremic serum compared to control serum, more prominently at 24 h and 40 h post-exposure. Figure 1D,E shows rates of oxygen consumption (OCR) in 3T3L1 cells exposed to uremic serum. In concordance with the increased lipolysis, cells exposed to uremia show increased basal respiration and proton leak rates. The increase in OCR in 3T3L1 cells exposed to uremia may be related to an increase in free fatty acid oxidation as expression of carnitine palmitoyltransferase I alpha (CPT-1α) is increased (Fig. 1F). The activity of CPT-1α is rate-limiting in fatty acid oxidation. These data demonstrate a significant change in adipose cell endocrine function and metabolism in uremia with increased production of inflammatory cytokines and lipolysis.

### Monocyte/macrophage cytokine production is heightened after uremia exposure

**Figure 2:**
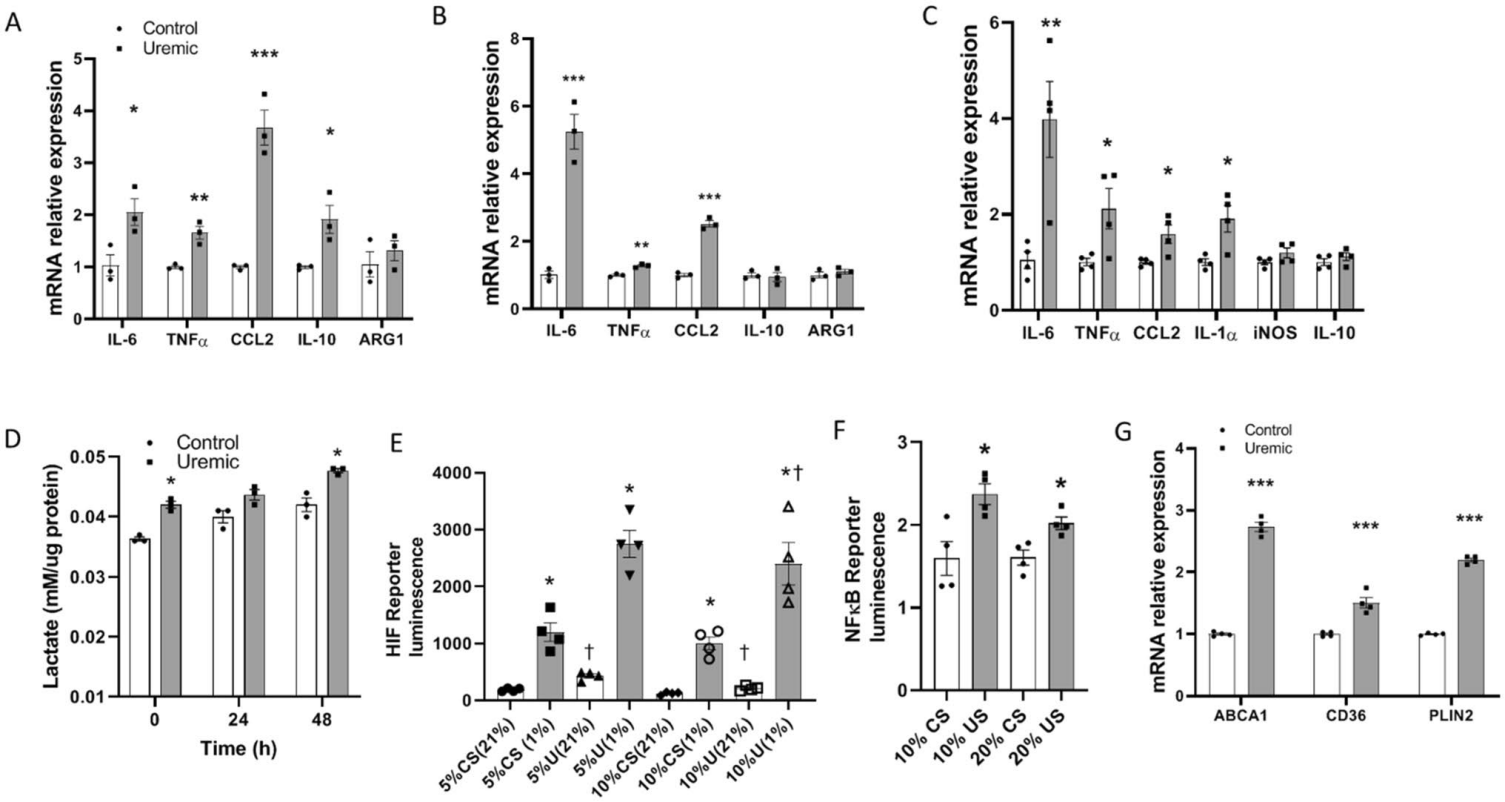
Monocyte/macrophage cytokine production is heightened after uremia exposure. (**A**) mRNA relative expression of cytokines in THP-1 monocytes cultured with 10% uremic versus 10% control serum. (**B**) mRNA relative expression of cytokines in HL-60 monocytes cultured with 10% uremic versus 10% control serum. (**C**) mRNA relative expression of cytokines in THP-1 macrophage cells after differentiation with PMA and treatment with 10% uremic versus 10% control serum. (**D**) Lactate levels in THP-1 cell supernatants after 24 h treatment with 20% control and uremic serum. Time 0 h uses supernatants of cells with serum treatment after 24 h. Time 24–48 h uses supernatant from cells cultured in standard media with calf serum. (**E**) NIH/3T3 cells expressing HIF luciferase reporter were incubated with different concentrations of normal and uremic serum. Luciferase activity as reflection of HIF activation in reporter cells was measured after serum exposure for 24 h in normoxia (21% O_2_) or hypoxia (1% O_2_). * compares each serum treatment in normoxia versus hypoxia, ^†^ compares each oxygen concentration in uremic versus normal serum treatment. (**F**) NIH/3T3 cells expressing NFκB luciferase reporter were incubated with different concentrations of normal and uremic serum. Luciferase activity as reflection of NFκB activation in reporter cells was measured after serum exposure for 24 h. (**G**) mRNA relative expression analysis of metabolic macrophage markers in THP-1 monocytes treated with PMA and exposed to 10% uremic and normal serum. Unpaired t test, n = 3 for mRNA experiments in (**A**–**C**), n = 4 for lactate measurements, luciferase activity and mRNA experiment in (**D**–**G**), experiments were done in triplicate, error bars represent SEM *^†^p ≤ 0.05, **p < 0.01, ***p < 0.001. CS, normal serum; US, uremic serum.

Since uremia alters adipose tissue cytokine production and increases ATMS infiltration^14^, we studied the effects of uremia on macrophage function by investigating their cytokine production after exposure to uremic serum. Human THP-1 monocytes incubated with uremic serum significantly increased expression of *IL-6, TNFα, CCL2* and, to a lesser extent, *IL-10* without any changes in the expression of arginase 1 (*ARG1*) (Fig. 2A). Comparable results were obtained using the HL60 cell line and THP-1 macrophages treated with phorbol 12-myristate 13-acetate (PMA) to induce macrophage differentiation. Both cell lines exposed to uremia have also a significant increase in the expression of *IL-6, TNFα, and CCL2* without changes in expression of anti-inflammatory cytokines (Fig. 2B,C). Since classically activated macrophages significantly rely on glycolytic metabolism, we studied lactate production in THP-1 cells exposed to uremic serum. As seen in Fig. 2D, lactate production is increased in macrophages exposed to uremic serum. The elevated glycolysis associated with inflammatory macrophages is heavily dependent on hypoxia-inducible factor-1 alpha (HIF-1α) which is reproduced in our uremic model. NIH/3T3 cells exposed to uremia demonstrated higher HIF-1α activity consistent with the elevated lactate production (Fig. 2E). Nuclear factor kappa-light-chain-enhancer of activated B cells (NFκB) is a key inflammatory transcription factor and as such, NIH/3T3 cells exposed to uremic serum demonstrate higher NFκB activation (Fig. 2F). We also studied macrophage markers associated with a metabolically activated phenotype^23^. THP-1 cells treated with PMA and then exposed to uremia, showed increased *ABCA1, CD36,* and *PLIN2* expression compared to macrophages exposed to normal serum (Fig. 2G). Similar results were found in THP-1 and HL-60 monocytes with increased expression of *PLIN2* and *CD36* (data not shown). Taken together, the data demonstrate that macrophage exposure to uremic serum promotes a change to an inflammatory phenotype with increased glycolysis and activation of HIF-1α and NFκB. Macrophages exposed to uremia also demonstrate increased expression of CCL2, an important macrophage-attractant chemokine as well as surface markers associated with a metabolically activated macrophage phenotype.

### Activation of HIF and NFκB in uremia is blunted by inflammatory cytokine inhibition

**Figure 3:**
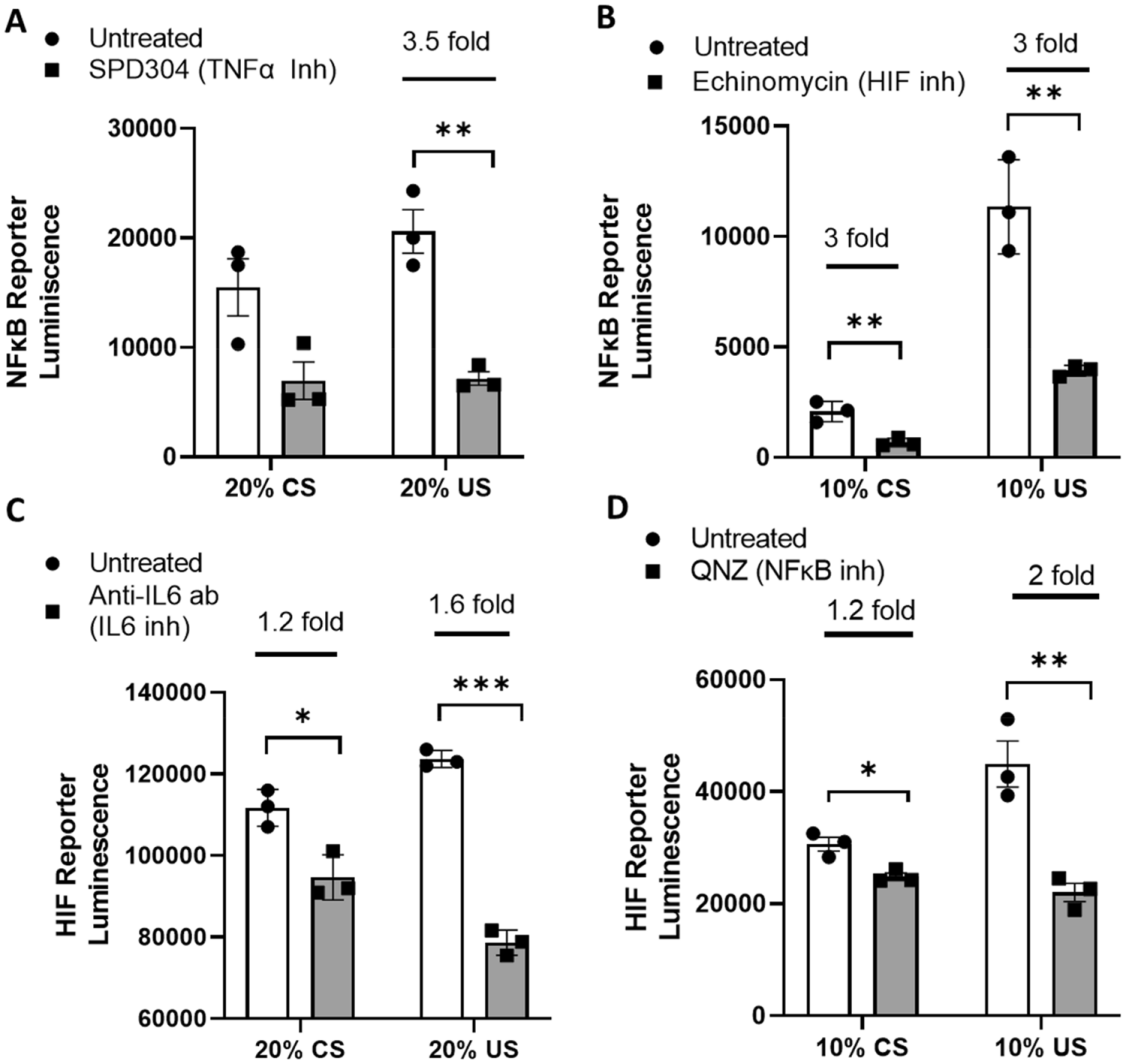
Activation of HIF and NFκB in uremia is blunted by inflammatory cytokine inhibition. NIH/3T3 cells expressing NFκB luciferase reporter were incubated with different concentrations of normal and uremic serum and 5 µM of the TNFα antagonist SPD304 (**A**) or 10 nM of echinomycin which is a HIF-1α inhibitor (**B**). Luciferase activity as reflection of NFκB activation in reporter cells was measured after serum and drug exposure for 24 h. NIH/3T3 cells expressing HIF luciferase reporter were incubated with different concentrations of normal and uremic serum and 2 µg/ml of neutralizing anti-IL6 monoclonal antibody (**C**) or 50 nM of QNZ which is an NFκB inhibitor (**D**). Luciferase activity as reflection of HIF activation in reporter cells was measured after serum exposure for 24 h in normoxia (21% O_2_). Unpaired t test, n = 3. Experiments were done in triplicate, error bars represent SEM *p ≤ 0.05, **p < 0.01, ***p < 0.001. CS, normal serum; US, uremic serum.

To determine the contribution of inflammatory cytokines to activation of HIF and NFκB, NIH/3T3 cells exposed to uremia were treated with IL6 and TNFα inhibitors. As shown in Fig. 3A, TNFα inhibition significantly decreases activation of NFκB in cells treated with uremic serum. No changes in NFκB activation were seen with IL-6 inhibition (data not shown). Conversely, IL-6 inhibition results in a significant decrease in HIF activation in cells exposed to uremic serum (Fig. 3C) whereas TNFα inhibition did not have any effects on HIF activation (data not shown). Moreover, HIF inhibition decreased NFκB activation and NFκB inhibition decreased HIF activation (Fig. 3B,D). Our data demonstrate that IL6 and TNFα contribute to the activation of HIF and NFκB respectively in uremia. There is also a feed-forward loop between NFκB and HIF in uremia.

### Adipocyte expression of cytokines is increased after exposure to uremic macrophages in co-culture

**Figure 4:**
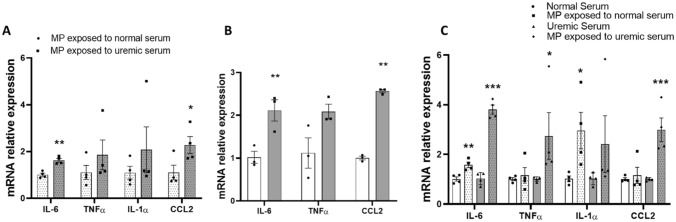
Adipocyte expression of inflammatory cytokines is increased after exposure to uremic macrophages in co-culture. (**A**) mRNA relative expression of cytokines in human subcutaneous adipocytes co-cultured with THP-1 macrophages exposed to uremic and normal human serum. (**B**) mRNA relative expression of cytokines in murine 3T3-L1 cells co-cultured with RAW 264.7 macrophages exposed to uremic and control human serum. (**C**) mRNA relative expression of cytokines in human subcutaneous adipocytes after co-culture with THP-1 macrophages treated with normal and uremic serum compared to serum exposure alone. Unpaired t test, n = 4 for (**A**) and (**C**), n = 3 for (**B**), experiments were done in triplicate, error bars represent SEM *p ≤ 0.05, **p < 0.01, ***p < 0.001. MP, macrophages.

In human obesity, interactions between macrophages and adipose tissue cells are known to enhance inflammation^24^. To better delineate the cross talk between adipose tissue and macrophages in uremia we used a co-culture model. Human and murine macrophages were exposed to uremic sera in separate inserts and then co-cultured with adipocytes in standard media. Adipose cells and macrophages were not physically in contact, but the insert pores allow the transfer of soluble factors. The cytokine production profile of adipose cells co-cultured with uremia exposed macrophages was then examined. Adipose cells exposed to uremia-primed macrophages showed increased *IL-6* and *CCL2* expression in the human (Fig. 4A) and murine (Fig. 4B) models. Expression levels of *IL1-α* in the murine model were too low to quantify (data not shown). Furthermore, expression of *IL-6, TNFα,* and *CCL2* was significantly higher in primary adipocytes co-cultured with uremic serum-exposed THP-1 macrophages compared to adipocytes exposed to uremic serum alone without macrophages (Fig. 4C). In an alternative experiment, macrophages without uremic exposure were co-cultured with adipocytes previously exposed to uremic serum. In this reverse condition, macrophages did not demonstrate changes in cytokine expression compared to macrophages co-cultured with adipocytes that were exposed to normal serum (data not shown).

### Increased adipose tissue macrophages (ATMS) in uremic mice present an inflammatory phenotype and IL-6 is a necessary cytokine for ATMS recruitment in uremia

**Figure 5:**
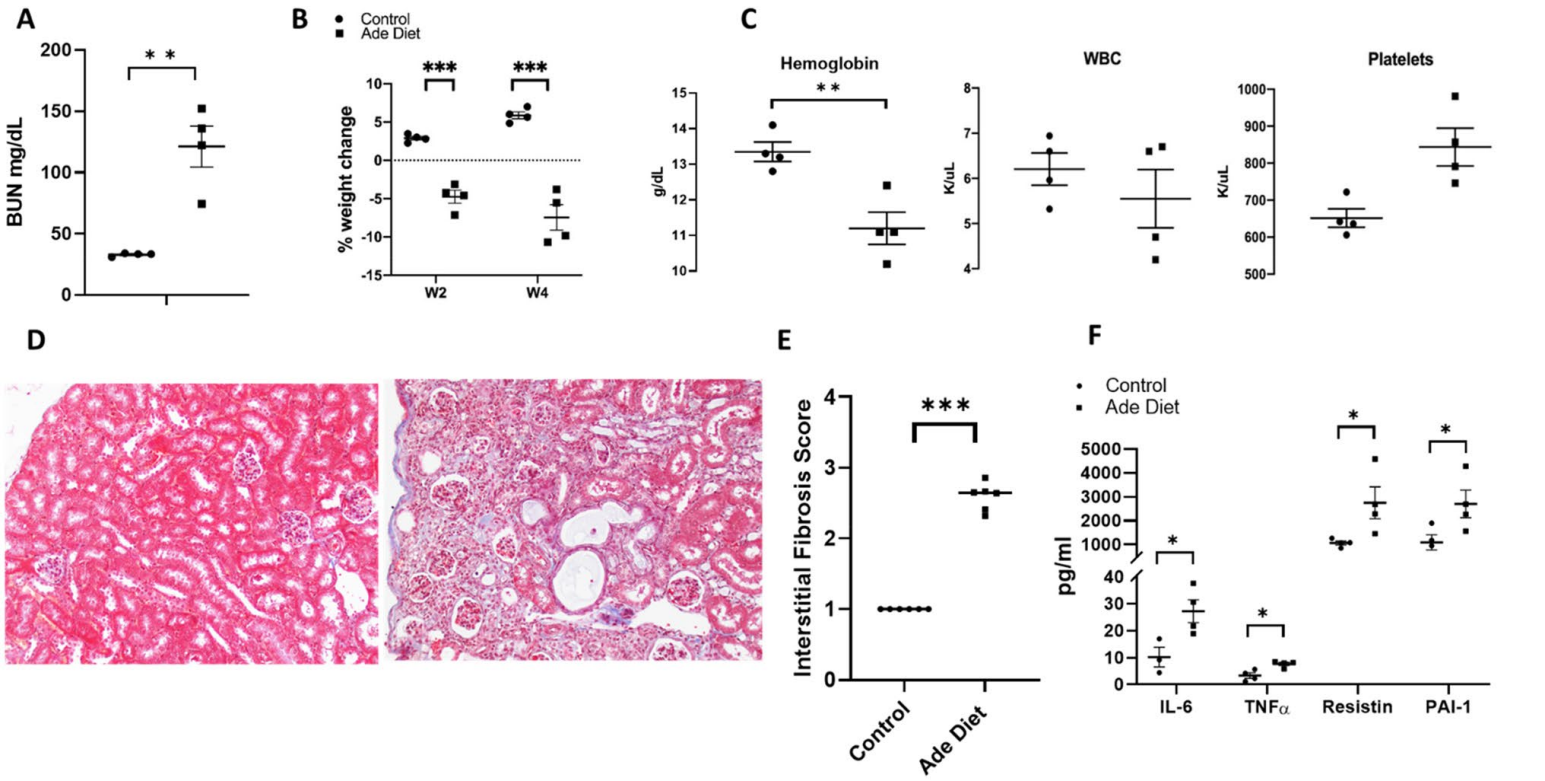
Phenotypic characteristics of adenine diet induced chronic kidney disease mice. (**A**) BUN level (mg/dL) in WT adenine diet fed mice versus control mice. (**B**) Difference in % of weight change at week 2 and week 4 of treatment between control and adenine diet treated mice. (**C**) Difference in hemoglobin (g/dL), white blood cells (K/μL), and platelets (K/μL) between the control group and the adenine diet group. (**D**) Representative photographs of sections of Masson Trichrome stained renal tissue from 2 animals treated with control or adenine diet for 4 weeks. Left panel of control animal shows preserved tubules, whereas the right panel demonstrates atrophic tubules with inflammatory interstitial infiltrate, (**E**) Interstitial fibrosis score of control and adenine diet groups on Masson Trichrome stained sections. At least 10 fields per mice were used at 20× magnification. (**F**) Comparison of cytokine values between the control group and the adenine diet group. Unpaired t test, n = 4, error bars represent SEM *p ≤ 0.05, **p < 0.01, ***p < 0.001. BUN, blood urea nitrogen. WT, wild type. Ade, adenine diet. WBC, white blood cells. PLT, platelets.

To determine if the uremic serum-induced changes seen in the human tissue and cell models could be recapitulated in-vivo, we used a mouse model of uremia by adding adenine to their diet. Baseline weight of control mice was 27.7 g and 29.7 g prior to adenine exposure in the experimental group. Mice exposed to adenine showed characteristics of advanced CKD including elevation of blood urea nitrogen (BUN), marked weight loss, development of anemia and higher platelet levels (Fig. 5A–C). Adenine induced changes in kidney histology included interstitial fibrosis, tubular atrophy, and 2,8-dihydroxyadenine crystals (Fig. 5D,E). Adenine exposed mice also have elevation of plasma inflammatory cytokines including IL-6, TNFα, resistin, and plasminogen activator inhibitor-1 (PAI-1) (Fig. 5F).

**Figure 6:**
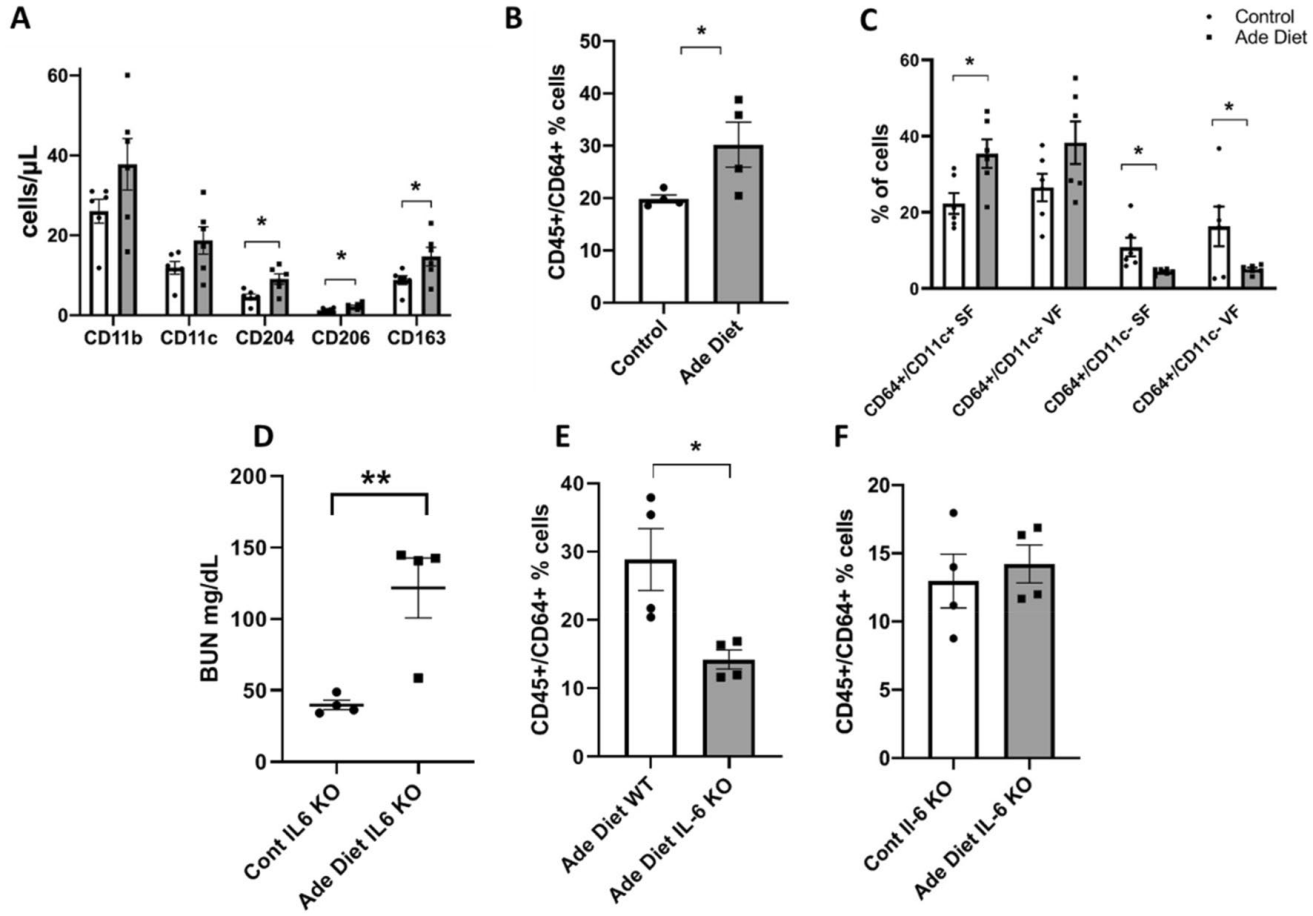
Peripheral and adipose tissue macrophage characteristics in uremia. (**A**) Peripheral blood flow cytometry showing the expression of different macrophage markers in adenine diet-induced mice versus control. (**B**) Adipose tissue flow cytometry showing the % of CD45+/CD64+ cells (macrophages) in adenine diet mice versus control mice. (**C**) Difference in % of expression of CD64+/CD11c+ and CD64+/CD11c- macrophage phenotypes in subcutaneous and visceral fat. (**D**) BUN level (mg/dL) in IL6 KO mice after adenine diet versus IL6 KO control mice. (**E**) Percentage of CD45+/CD64+ cells in adenine diet treated wild type and IL-6 KO mice; (**F**) Percentage of CD45+/CD64+ cells in IL-6 KO mice treated with adenine diet versus control. Unpaired t test, n = 4 for (**B**), (**D**), (**E**), n = 6 for (**A**), (**C**), error bars represent SEM *p ≤ 0.05. SF, subcutaneous fat. VF, visceral fat. Ade, adenine diet. Cont, control. KO, knockout.

To investigate macrophage characteristics in our mouse model, we initially examined monocyte surface marker expression in peripheral blood. Adenine exposed mice had an elevated percentage of circulating monocytes with higher numbers of cells bearing the surface markers CD11b (a pan-macrophage marker), CD11c (marker of classically activated macrophages), CD204, CD206, and CD163 (all three are markers of alternatively activated macrophages) (Fig. 6A). These data suggest a distinct uremic peripheral monocyte phenotype with an increase in both M1 and M2-like monocyte/macrophage subtypes.

We then determined the phenotype of ATMS in uremic mice by flow cytometry. Uremic mice have a higher percentage of CD45^+^/CD64^+^ cells compared to control mice as shown in Fig. 6B. When stratified by surface markers commonly associated with classically activated macrophages, versus alternatively activated macrophages, subcutaneous fat (SF) depots show a preponderance of classically activated macrophages with a lower proportion of alternatively activated macrophages in both SF and visceral fat (VF) (Fig. 6C).

Since IL-6 is the cytokine with higher expression changes in the in-vitro adipose tissue cell and macrophage models, we hypothesize that IL-6 has a leading role in macrophage recruitment to adipose tissue in uremia. We then compared the percentage of CD45^+^/CD64^+^ cells in uremic wild type mice compared to uremic IL-6 knock out (KO) mice. IL-6 KO mice were exposed to adenine supplementation in the diet and developed a similar phenotype to the uremic wild type counterparts (Fig. 6D). However, uremic IL-6 KO mice have reduced CD45^+^/CD64^+^ cells in adipose tissue compared to their uremic wild type counterparts (Fig. 6E). In contrast to wild-type control mice, there was no significant difference in the percentage of ATMS between uremic IL-6 KO mice and IL-6 KO mice with normal kidney function (Fig. 6F). The data from the in-vivo mouse models of uremia are consistent with prior literature describing an increase in ATMS in ESRD patients compared to controls with normal kidney function^14^. The data is also consistent with our in-vitro uremic models as more of the ATMS in the uremic mice have an M1-like phenotype than an M2-like phenotype. The data also supports a possible mechanism of macrophage infiltration in uremia. Our data postulates that IL-6 is a key cytokine regulating macrophage recruitment to adipose tissue in uremia.

### Uremic patients have increased adipose tissue macrophage numbers than controls independently of BMI

Since there is a positive correlation between ATMS infiltration and BMI in humans with normal kidney function^25^, we set out to determined if there are differences in ATMS infiltration in uremic patients versus controls after BMI stratification.

The demographic characteristics of the ESRD and control cohorts are described in Table 1. Diabetic patients were intentionally excluded from the study to minimize the confounding of adipose tissue changes related to metabolic disorders in addition to uremia. There were more obese patients in the ESRD group than the controls as expected, but median weight was similar between the groups.

**Table 1:** Demographic characteristics of the study participants.

Demographics	Controls (N = 36)		ESRD (N = 41)	
Age (years) median	47 (29, 53)		54 (43, 61)	
**BMI (kg/m**^**2**^**) = N; median**				
Normal weight (BMI < 25)	N = 13	23.2 (20.57, 23.60)	N = 13	22.8 (19.9, 23.9)
Overweight (BMI = 25–29.9)	N = 15	26.9 (26.39, 28.8)	N = 15	27.3 (26.04, 28.55)
Obese (BMI > 30)	N = 8	32.66 (31.66, 33.1)	N = 13	33.7 (32.9, 34.7)
Height (m) median	1.69 (1.65, 1.75)		1.72 (1.61, 1.77)	
Weight (kg) median	80.27 (69.61, 87.64)		80.04 (69.84, 88.89)	
Sex (female)	22 (61.1%)		20 (48.8%)	
**Race**				
Black or African American	3 (8.3%)		7 (17%)	
White	32 (88.9%)		32 (78%)	
Other	1 (2.8%)		2 (5%)	
**Ethnicity**				
Hispanic	2 (5.5%)		3 (7.3%)	
Non-Hispanic	34 (94.5%)		38 (92.7%)	
**Cause of CKD**				
HTN	NA		13 (31.7%)	
GN	NA		14 (34.1%)	
PKD	NA		3 (7.3%)	
Other	NA		11 (26.8%)	
Previous transplant (yes)	NA		6 (14.6%)	
**Renal replacement modality**				
HD	NA		21 (51.2%)	
PD	NA		5 (12.2%)	
Preemptive	NA		15 (36.6%)	
Albumin (g/dl) median	4.6 (4.5, 4.8)		4.5 (4.3, 4.6)	

Age, BMI, height, weight, and albumin are represented as median (Q1, Q3).

All categorical data is presented as number (percent).

ESRD, end stage renal disease; BMI, body mass index; HTN, hypertension; GN, glomerulonephritis; PKD, polycystic kidney disease; HD, hemodialysis; PD, peritoneal dialysis.

Percentage were rounded to nearest decimal point.

**Figure 7:**
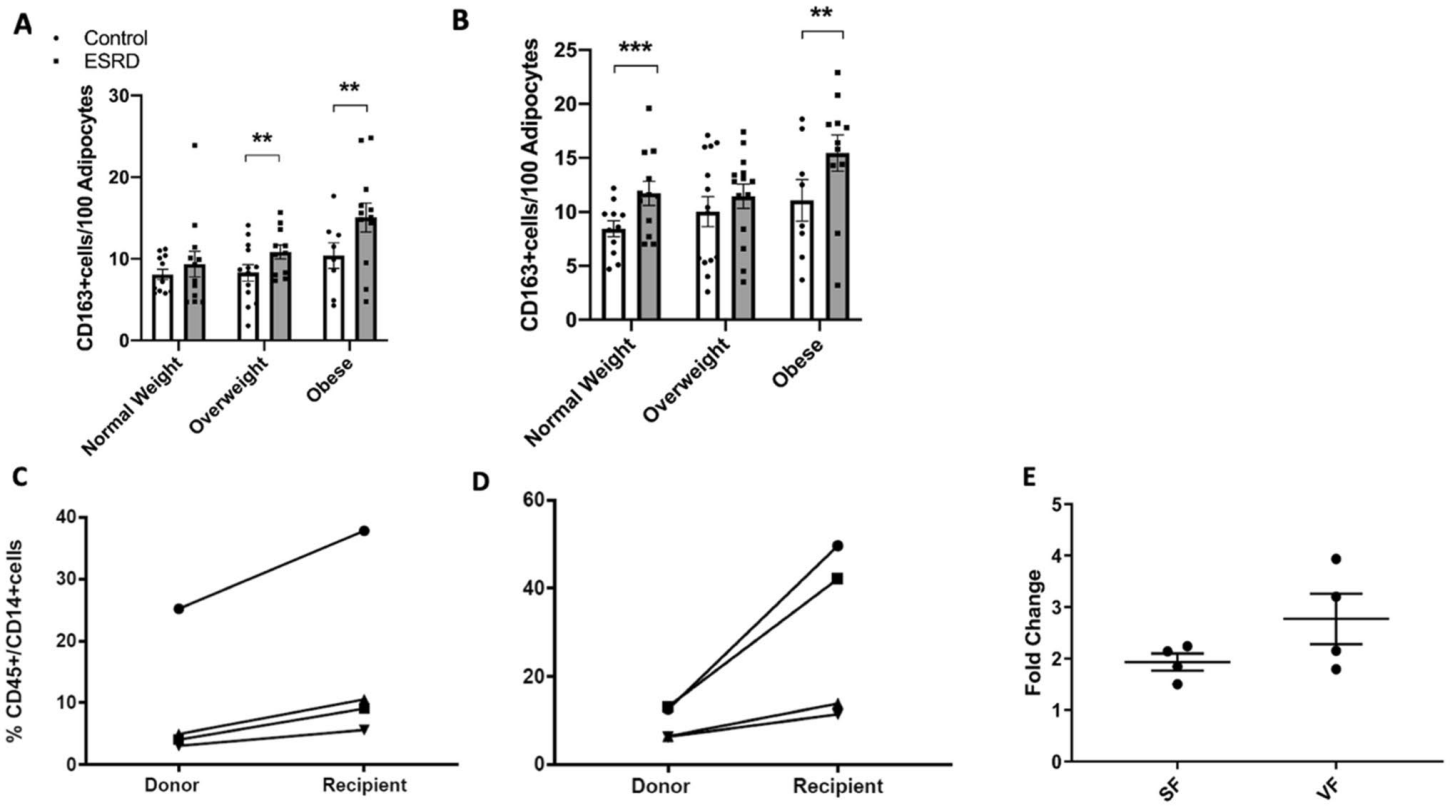
Adipose tissue macrophage counts in uremic patients versus controls stratified by BMI. (A) Mean number of CD163+ cells per 100 adipocytes in subcutaneous adipose tissue. (**B**) Mean number of CD163+ cells per 100 adipocytes in visceral adipose tissue. (**C**) CD45+/CD14+ % cells (macrophages) in subcutaneous adipose tissue of kidney donors compared to kidney transplant recipients (n = 4 per group). (**D**) CD45+/CD14+ % cells in visceral adipose tissue (of kidney donors compared to kidney transplant recipients. Lines link participant pairs (donor and recipients) that were analyzed on the same day (**E**) Macrophage fold change between donors and recipients in subcutaneous versus visceral fat. Mann–Whitney U test, error bars represent IQR. *p ≤ 0.05. SF, subcutaneous fat. VF, visceral fat. ESRD, end stage renal disease.

There was an increase in macrophage infiltration using CD163 immunostaining as a pan-macrophage marker in both SF and VF in ESRD participants compared to controls (data not shown) and this was maintained after stratifying by BMI in almost all groups (Fig. 7A,B). Data was replicated by flow cytometry of stromal vascular fraction (SVF) in uremic patients and controls showing higher percentage of CD45+/CD14+ cells in ESRD patients compared to controls (Fig. 7C,D). Specifically, SF of ESRD patients had approximately double the number of macrophages than controls and approximately 3 times more macrophages in VF than controls (Fig. 7E). Since our in vitro and in-vivo model demonstrated an inflammatory phenotype in macrophages exposed to uremia, we then hypothesized that ATMS in uremic patients have a different phenotype than macrophages in non-uremic conditions. Because cell size and morphology have been used to identify macrophage functional phenotypes^26,27^, we used macrophage morphology as a surrogate of phenotype. Macrophage morphology was assessed by measuring macrophage non-nuclear fractions as a marker of macrophage area. Non-nuclear fractions were defined as areas that had positive CD163 staining but were not associated with nuclei. When macrophage nuclei and fractions were quantified, macrophage number and area were higher in both of the adipose tissue depots and in every strata of BMI (Supplemental Figures 1,2). Adipocyte size did not differ between ESRD participants and controls in SF (data not shown). In VF, adipocytes were larger in both lean and overweight ESRD patients compared to controls (Supplemental Figure 3).

Our human tissue data demonstrate an increase in macrophage recruitment to adipose tissue in uremia that is independent of adiposity. ESRD also drives a distinct adipose tissue macrophage phenotype with increased macrophage area.

## Discussion

Our findings demonstrate that adipocytes and macrophages contribute to uremia-induced inflammation. Our data show that adipocytes and macrophages exposed to uremia increase inflammatory cytokine secretion and activation of NFκB/HIF1α. The uremic environment also promotes macrophage recruitment demonstrated by increased *CCL2* expression in macrophages. We also demonstrated a positive interaction between uremia-primed macrophages and adipocytes. Adipocytes exposed to uremia-primed macrophages express greater inflammatory cytokine secretion than after exposure to uremic sera alone. In uremic mice, there is greater ATMS density than controls; and ATMS in uremic mice predominantly display an inflammatory phenotype. IL-6 abrogation prevents recruitment of ATMS in uremic mouse models. In uremic human adipose tissue, ATMS density is greater compared to normal kidney function controls, independent of BMI.

In our current study, we present data supporting the concept that uremia stimulates adipocyte production of inflammatory cytokines and promotes lipolysis. This finding is consistent with prior studies that reported increased IL-6 and TNF-α expression in 3T3-L1 adipocytes exposed to uremic toxins such as indoxyl sulfate and p-cresyl sulfate^28,29^. Furthermore, we have also demonstrated that macrophages exposed to uremia drive adipocytes to produce inflammatory cytokines. Uremia is a complex metabolic state seen in advanced kidney disease that results from accumulation of multiple toxic compounds including degradation products of protein metabolism, cytokines, interleukins, ions such as hydrogen, advanced glycation end products, and other metabolites, as well as changes in redox state that negatively affect organ function. The contribution of each component of the uremic environment that drives adipose tissue metabolic changes will need to be determined in the future with studies of adipose tissue metabolism after exposure to increasing concentrations of uremic toxins. Interestingly, despite production of inflammatory cytokines, adipocytes exposed to uremia also increase production of adiponectin. We previously demonstrated higher adiponectin production in adipose tissue of patients with advanced CKD compared to controls^1^, and in this study, we observed similar results in an adipocyte cell model exposed to uremia. Adiponectin function in uremia appears to be blunted, as demonstrated by muscle cell adiponectin resistance at the post-receptor level^30^ and by increased lipolysis with decreased lipogenesis in adipocytes exposed to uremia^31,32^. Elevated adiponectin production in uremia could be a compensatory response to the increased inflammatory condition, worsening insulin resistance or decreased adiponectin function. Similarly to other groups, we have demonstrated that adipose tissue cells exposed to uremic serum demonstrate higher lipolysis rates^31^. But in contrast to obesity, adipose tissue cells exposed to uremic serum present higher basal oxygen consumption rates reflecting a shift to energy production and increased basal metabolic rate (BMR). Higher BMR in adipose tissue may reflect a response to the enhanced energy expenditure that accompanies chronic inflammation and advanced kidney disease undergoing renal replacement therapy. Further studies will need to elucidate if the increase in OCR reflects increase in FAO or mitochondrial uncoupling and the factors that drive this metabolic shift.

Circulating monocytes are other important sources of inflammatory cytokines. Reports about the monocyte/macrophage phenotype in uremia are limited. Using surface markers associated with an M1–like phenotype, Merino et al.^33^. and Bonan et al.^34^. described an increase in the proportion of pro-inflammatory circulating monocytes in uremic patients. Functional studies on cytokine expression and macrophage adhesion/migration properties in response to uremia are also limited to a few reports. Adesso et al.^35^. determined that the uremic toxin indoxyl sulfate enhances production of IL-6 and TNFα in macrophages also exposed to bacterial lipopolysaccharide. However, no description was provided on functional changes in macrophages with uremia exposure alone. In cell culture studies, macrophages responded to uremic serum exposure with increased migration and adhesion to endothelial cells^36^. These early reports indicate that uremia changes macrophage phenotype. Our data is consistent with and adds to prior literature^37^, as we demonstrate that uremic serum exposure increases production of inflammatory cytokines and the important macrophage chemotactic protein CCL2 in monocyte and macrophage human cell lines. Macrophages exposed to uremic serum have higher lactate production, reflecting a switch to glycolysis, which has been described as a key feature of classically activated macrophages^38^. 3T3/NIH cells exposed to uremia had NFκB and HIF1α activation highlighting a role of both transcription factors in the inflammatory response of uremia. Upregulation of HIF1α and NFκB genes in uremic patients have been described in peripheral blood mononuclear cells^39^ Our data showing activation of both transcription factors along with an increase in lactate release is compatible with the known existence of a positive feedback loop between NFκB and HIF1^40,41^. Furthermore, we have demonstrated that IL6 plays a key role on HIF activation and TNFα in NFκB activation in uremia. A feed-forward loop exists between NFκB and HIF activation as NFκB drives HIF activation and HIF drives NFκB activation.

Moreover, uremia exposure induced expression of markers associated with a metabolically activated phenotype. Our findings indicate that uremia contributes to both activation and polarization of macrophages to a metabolic phenotype promoting inflammation as well as macrophage migration to affected organs, in our case adipose tissue. Taken together, these data demonstrate that macrophages play a key role in uremic inflammation. Further studies in all tissues in uremia are needed to determine if the inflammatory macrophage phenotype is a global phenomenon or is restricted to a tissue subset, which includes the adipose tissue.

Our data demonstrate a significant interaction between adipose tissue cells and macrophages in uremia as adipocytes express greater inflammatory cytokine production following exposure to macrophages primed by uremic serum. While exposure to uremic serum alone promotes an inflammatory response in adipocytes, exposure to uremic macrophages promotes a much stronger adipocyte inflammatory response.

In contrast to adipose tissue in obesity, macrophages exposed to uremic adipocytes do not undergo an inflammatory switch. Our data highlight a significant difference between the pathogenesis of the adipose tissue inflammation in uremia versus obesity as it demonstrates that the macrophage inflammatory switch is primarily mediated by exposure to uremic serum and not to uremic adipocytes. Recently, lipid filled vesicles released by adipocytes have been described as a potential mechanism of ATMS differentiation^42^. Further studies are needed to characterize the role of adipose tissue lipid release and ATMS differentiation in uremia.

The contribution of adipose tissue to the increased inflammatory state seen in kidney disease has been recently highlighted by Sodhi et al.^43^. Using a partial nephrectomy model in mice, the study demonstrates increased production of systemic inflammatory cytokines by adipocytes that worsens with high fat diet. The authors also propose that alterations of the redox state in adipocytes play a role in the pathogenesis of uremic adipocyte dysfunction. Our study is consistent with the published data as our mice model of kidney disease after adenine supplementation showed increased peripheral inflammatory cytokines. On the other hand, we focused on ATMS content as a potential pathway for adipocyte dysregulation. The macrophage content in adipose tissue of non-obese humans can vary between 1 and 30%. In obesity, the content of ATMS can double, contributing to low-grade chronic inflammation and adipose tissue dysregulation^16^. In this study, we used a mouse model to study ATMS in uremia. Our uremic mice demonstrated higher peripheral monocyte numbers compared to control mice and consistent with human peripheral monocyte studies with a greater percentage of CD14^+^/CD16^+^ monocytes in dialysis patients compared to healthy controls^33,34^. In contrast to the human findings, peripheral monocytes in uremic mice express a greater variety of cell surface markers including markers associated with classically and alternatively activated macrophages, which may be due to lack of discrimination of M1 and M2 markers for metabolically activated macrophages^23^. In human obesity, recruitment of peripheral monocytes and local proliferation of resident macrophages contribute to the increased numbers of ATMS^44,45^. The source of increased ATMS in uremia needs to be determined but higher numbers of circulating peripheral monocytes in uremia as well as elevated CCL2 and IL-6 in the cell models suggest a preferential role of monocyte recruitment to adipose tissue.

Uremic mice demonstrate higher ATMS density compared to control mice and macrophages in uremic adipose tissue show predominantly a classically activated phenotype. The macrophage phenotype in our mouse model is consistent with the inflammatory phenotype found in our cell culture experiments and is consistent with the phenotype described in the obesity literature^18^, reinforcing the potential contribution of adipose tissue to the uremic inflammatory condition.

In uremic patients, among a variety of inflammatory biomarkers, IL-6 has been demonstrated to be a robust predictor of cardiovascular disease and all-cause mortality^46^. Besides its role as a biomarker in CKD, IL-6 signaling leads to recruitment of monocytes to sites of inflammation^47^. Our uremic mouse model demonstrates that IL-6 plays a key role in macrophage recruitment to adipose tissue. IL-6 KO uremic mice had lower ATMS density when compared to wild type uremic mice. There were also no differences in ATMS density in uremic IL-6 KO mice compared to non-uremic IL-6 KO mice highlighting the necessary role of IL-6 in macrophage infiltration in uremia. Because ATMS can contribute up to 50% of the production of circulating IL-6^25^ it is possible that a decrease in adipose tissue macrophage infiltration in uremic mice could potentially result in a decrease in the overall inflammatory state of uremia.

Our study provides additional findings on adipose tissue as a source of inflammation in uremic patients. We demonstrated greater macrophage density in different BMI strata in visceral and subcutaneous adipose tissue depots in ESRD patients compared to controls, indicating that macrophage infiltration in uremia is unrelated to the degree of adiposity. Our data are consistent with findings by Gertow et al.^48^ who found more CD68^+^ cells in subcutaneous adipose tissue of non-obese uremic patients compared to healthy controls. The authors also described smaller adipocytes in patients with CKD. On the other hand, our ESRD patients did not have significant differences in adipocyte size in subcutaneous adipose tissue while adipocytes were larger in visceral adipose tissue of non-obese uremic patients. The discrepancy could be explained by our strict BMI matching between groups and by the separation of the lean and overweight sub-groups. Visceral fat is considered the more functional adipose depot and larger adipocytes in uremic patients could be a reflection of altered adipocyte metabolism associated with uremia.

Our study has some limitations. Macrophage characterization in our tissue experiments was based on standard surface markers generally associated with classically activated versus alternatively activated macrophage phenotypes. Recently, there has been additional characterization of macrophages in metabolic diseases such as obesity and other disorders^23^. Functional as well as phenotypic characterization of tissue macrophages should be considered in vivo uremic models as it is possible that uremia-primed macrophages have a unique phenotype. We utilized an adenine nephropathy mouse as a model of advanced kidney disease. Further studies to explore the role of our findings will require replication in different mouse models of uremia. Although our study focused on the role of IL-6 in ATMS recruitment, other inflammatory cytokines and uremic toxins may also play a role in macrophage recruitment and will need to be investigated.

Uremic patients have clinical evidence of chronic inflammation and severe uremic inflammation is predictive of adverse outcomes. Uremia promotes changes in both adipose tissue and macrophages in a cooperative fashion that enhance the inflammatory condition. Although the specific contribution of adipose tissue to uremic inflammation remains unclear, our co-culture studies demonstrate an interaction between adipocytes and macrophages that drives additional adipocyte inflammation. Findings in both our uremic mouse model and human tissue are consistent with the overall concept that interplay between uremia activated macrophages and adipose tissue augments the inflammatory process in uremia. The role of uremia-primed macrophages driving tissue dysfunction in non-adipose tissues will need to be determined in the future.

## Methods

### Cell culture

3T3-L1 cells were used as a model of murine adipocytes. THP-1 monocyte-like cells and HL-60 promyeloblasts were used as human monocyte cell lines and were maintained in suspension. THP-1 cells can be differentiated to adherent macrophages by using Phorbol-12-mystrated-13-acetate or PMA. RAW 264.7 cells were used as a murine macrophage cell line. All cell lines were purchased from ATCC and cultured according to the manufacturer’s recommendations. Briefly, 3T3-L1 cells were cultured in 10% bovine calf serum (ATCC 30-2030) supplemented DMEM. When confluent, cells underwent a pre-adipose to adipose like conversion by replacing medium to DMEM containing 10% fetal bovine sum (FBS), 1 µM dexamethasone (D4902; Sigma), 0.5 mM 3-isobutyl-1-methylxanthine (I5879; Sigma), and 1 µg/mL insulin (I0516; Sigma). 3T3-L1 adipocyte like cells were maintained in DMEM 10% FBS and 1 µg/mL insulin. The RAW 264.7 cells were maintained in 10% bovine calf serum supplemented DMEM. THP-1 human monocytic cells were maintained in RPMI-1640 Medium (ATCC 30-2001) supplemented with 10% FBS and 2-mercaptoethanol (21985023; Gibco) to a final concentration of 0.05 mM. Suspension THP-1 cells (500,000 cells per well) were differentiated into adherent plates using 75 ng/mL of PMA (P8139; Sigma) for 72 h. HL-60 human promyeloblast cells were maintained in Iscove's Modified Dulbecco's Medium (ATCC 30-2005) supplemented with 20% FBS.

Human pre-adipocytes (HPAd) were purchased from Sigma (802S-05A). Cells were grown and differentiated to human adipocytes (HAd) following manufacturer’s instructions. Briefly, 44,000 preadipocytes per cm^2^ were seeded and incubated until they reached 100% confluence. Confluent cells were cultured with Human Adipocyte Differentiation Medium (811D-250) for 15 days until cells had lipid droplets. One day prior to the assay, cells were starved in Human Adipocyte Starvation Medium (811S-250). Experiments with cell lines were performed within 6 months of purchase and cells had tested negative for mycoplasma before their use.

### Adipocyte—macrophage co-culture

3T3-L1 with RAW 264.7 and HAd with THP-1 were cocultured using a transwell system. Adipocytes were differentiated at the bottom of a 12-well plate and maintained in differentiation media with calf serum. Separately from the adipocytes, macrophages were seeded in 0.4 µm pore-size membrane inserts (353180; Corning) and treated with 10% normal or uremic human serum for 24 h. After serum treatment, media was washed, and inserts containing pretreated macrophages were placed over the adipocytes and cocultured in standard differentiation media for 24 h. The co-culture experiments were repeated with macrophages at the bottom of the 12-well plate and adipocytes exposed to uremia in the membrane inserts.

### Human serum treatment

For the experiments with uremic serum exposure, uremic serum was collected, prior to hemodialysis (HD) after the longest break (after 2 dialysis free days), from a pool of stable in-center hemodialysis patients. 10 mL of uremic serum were drawn from approximately 100 HD patients in their usual state of health (we excluded patients with Hepatitis C, Hepatitis B or acute infection). For the normal serum, 10 mL of normal serum were drawn from 50 kidney donors in their usual state of health before donation. Serum from dialysis patients and kidney donors was pooled, filtered, and aliquoted in small vials to be frozen at − 80 °C for cell experiments.

### Quantitative real time-PCR

Total RNA from the cell experiments was extracted using TRIzol reagent (15596026; Invitrogen). cDNA was obtained using the High-Capacity cDNA Reverse Transcription Kit (4368814; Applied Biosystems). Quantitative Polymerase Chain Reaction (qPCR) was performed in triplicates and performed using QuantStudio 12 K Flex Real-Time PCR System (Thermo Fisher Scientific). TaqMan Gene expression assays used for the experiments are detailed in Supplemental Table 1. Expression levels were calculated using the relative comparative CT (2^−∆∆Ct^) method after normalization to GAPDH as an endogenous control.

### Glycerol release assay

Differentiated 3T3-L1 cells were exposed to control or uremic serum in 12-well plates. After 24 h, cells were washed, and media replaced by fresh DMEM 10% FBS. The cell culture supernatants were collected after 24 h and 40 h. Released free glycerol was measured using the Glycerol Assay Kit (MAK117, Sigma-Aldrich) according to the manufacturer’s instructions. Results were normalized to protein concentration (µg/µL).

### Oxygen consumption rates (OCR) measurement

3T3-L1 cells were plated and differentiated on Seahorse XF 24-well plates (100777-004, Agilent). Cells were exposed to control or uremic serum for 24 h, and incubated in Seahorse XF DMEM medium (103334-100, Agilent) supplemented with 5 mM glucose (103577-100, Agilent) at 37 °C for 45 min without CO_2_ just before running the experiments. Seahorse XF Cell Mito Stress Test Kit (103015-100, Agilent) was used with the final respiratory modulators concentration of 1.5 µM for Oligomycin, 1 µM for FCCP, and 0.5 µM for Rotenone/Antimycin A. OCR measurements were obtained using the Seahorse XFe24 Analyzer, and normalized to protein concentration (µg/µL).

### Extracellular lactate measurement

THP-1 adherent cells were exposed to serum in 12-well plates. After 24 h, cells were washed, and media replaced by fresh RPMI 10% FBS. The cell culture supernatants were collected after 24 h and 48 h. Extracellular l-lactate concentration was measured using the EnzyChrom l-lactate Assay Kit (ECLC-100, BioAssay Systems) according to the manufacturer’s protocol. Results were normalized to total protein content (µg).

### Patients

Participants were recruited from the Thomas Jefferson University Hospital (TJUH) transplant program. The study protocol was approved by the Institutional Review Board at TJUH and informed consent was obtained from each participant. All methods were carried out in accordance with relevant guidelines and regulations (Declaration of Helsinki). Patients with diabetes pre-kidney transplantation were excluded. The control group consisted of kidney donors. While under general anesthesia for kidney donation or transplantation, 250 mg of omental visceral fat and subcutaneous fat were obtained. Recruitment of patients and procedures have been previously described by us^14^.

### Histology

Adipose and kidney tissues were paraffin embedded. Kidney sections of control and adenine diet mice were stained with Masson Trichrome in our pathology core. An interstitial fibrosis (IF) score was obtain using previously described criteria^49–51^ on at least 10 randomly selected areas at 20× magnification in in each mice. Scores were verified by 2 different investigators blinded to the study groups. Inter-observer variability was < 10%. In brief, IF of 1 represents an area with interstitial fibrosis of less than 25%, 2 represents IF of an area between 26 and 50%, 3 represents IF of an area between 51 and 75% and 4 represents IF of an area > 75%. Paraffin-embedded sections adipose tissue were immunostained as previously described by us with minor modifications^14^. Briefly, sections were deparaffinized, rehydrated and antigen retrieval was performed in 10 mM sodium citrate, pH 6.0 with 0.05% Tween-20 for 10 min using a pressure cooker. Sections were blocked with 3% hydrogen peroxide for 15 min followed by incubation using an avidin–biotin blocking kit (AB972, BioCare Medical) to block endogenous biotin. After incubation with 10% goat serum for 1 h, sections were incubated with primary antibody overnight at 4 °C (CD163 (MRQ-26), Sigma). Antibody binding was detected using a biotinylated secondary antibody (BA-9200, Vector) and the Vectastain ABC HRP Kit (PK-4000, Vector). Immunoreactivity was revealed using 3,3′ diaminobenzidine with a liquid DAB kit (K346811-2, Agilent Technologies). ATMS infiltration was measured as the number of CD163^+^ cells with nuclei in 10 randomly chosen fields per adipose tissue depot and participant at 40× magnification and evaluated by two independent blinded investigators. Non-nuclear contiguous CD163^+^ areas, referred to as non-nuclear fractions, were also counted. To evaluate the relative contribution of the non-nuclear fractions, we divided the number of fractions per image by 3, which best represented their smaller size, as compared to CD163^+^ staining with nuclei. The average adipocyte area was calculated for each image by dividing the image area, which was 95,000 square microns for all images, by the number of adipocytes in each image.

### Animal care and use

8 to 10 weeks old male C57BL/6 mice (Strain 000664) and B6.129S2-Il6tm1Kopf/J (Strain 002650) were obtained from Jackson Laboratories and fed a standard diet (PicoLab Rodent Diet 20, 5053) or 0.2% adenine-containing diet (Modified LabDiet PicoLab Rodent Diet 20, 5053, with 0.2% adenine A8626; Sigma) ad libitum for 4 to 6 weeks. After development of kidney disease, mice were euthanized by CO_2_ asphyxiation. After euthanasia, blood was obtained by intracardiac puncture and dorso-lumbar and epididymal fat pads were isolated. All animal procedures were in compliance with protocols approved by the Institutional Animal Care and Use Committee at Thomas Jefferson University. All the methods were carried out in accordance with relevant guidelines and regulations. The study was carried out in compliance with the ARRIVE guidelines.

### Blood immunophenotyping by flow cytometry (FCM)

Performed from retro-orbital blood. Briefly, from 10 µL of whole blood, red cells were lysed with ACK Lysing Buffer (A1049201; Gibco). Samples were washed with 1% BSA in PBS and incubated in Fc-block (antiCD16/32; 101302; BioLegend) on ice. Cell surface markers were stained at 4 °C with antibodies described in Supplemental Table 2. After cell fixation, 50 µL counting beads (C36950; Molecular Probes) were added for accurate cell counting. Cells were sorted on a BD LSR Fortessa cell analyzer and data analyzed using FlowJo v10.6 software.

### Adipose tissue macrophage flow analysis

Dorso-lumbar and epididymal fat pads of normal and uremic male mice (n = 4 per experiment) were used for characterization of ATMS. For human ATMS studies, 4 recipients of kidney transplant and 4 healthy kidney donors were included. Stromal vascular fractions were isolated and separated from adipocytes as described previously^52^. The SVF pellet was resuspended in 0.5 mL RBC Lysis Buffer (eBioscience) and then placed in FACS buffer (PBS with 1% heat inactivated FBS, 1 mM EDTA, and 25 mM HEPES). Cells were incubated in Fc-block (antiCD16/32) on ice and stained with indicated antibodies. For viability assessment, cells were suspended in FACS buffer containing DAPI (0.2 µg/mL). Antibodies for flow cytometry are provided in Supplemental Table 2. Cells were sorted on an LSR II Flow Cytometer (BD Biosciences) and data were analyzed using FlowJo 10.6 software.

### Western Blot

Protein extraction, quantification, and immunoblotting were performed as previously described^1^. Primary antibodies used include anti-alpha 1 Sodium Potassium ATPase (ab7671; Abcam), anti-adiponectin (2789; Cell Signaling Technology), and anti-β-actin (A5441; Sigma).

### Blood urea nitrogen (BUN)

Analyzed from murine retro-orbital blood with the QuantiChrom Urea Assay Kit (DIUR-100) as directed by the manufacturer.

### Complete blood count (CBC)

Analyzed in 50 µL of murine whole blood in a GENESISTM Veterinary Hematology System by the Translational Research/Pathology Core at the Sidney Kimmel Cancer Center.

### Cytokine analysis

Plasma IL-6, TNFα, insulin, resistin, and PAI-1 levels were measured using a Milliplex MAP Mouse Adipokine Magnetic Bead Panel (MADKMAG-71K; EMD Millipore) and analyzed by Luminex xMAP in a FLEXMAP 3D System.

### Luciferase activity

NIH/3T3 cells expressing NFκB luciferase reporter and HIF Luciferase reporter (RC0015 and LR0128, Panomics, CA) were seeded in 12-well plates with 1 × 10^5^ of cells per well. The next day, the media was changed to 5/10/20% normal serum or uremic serum in DMEM and cells were incubated for 24 h. 5 µM of SPD304 (S1697; Sigma) which is a TNFα antagonist; 2 µg/mL of neutralizing anti-IL6 monoclonal antibody (MAB406; R&D Systems), 10 nM of echinomycin (SML0477; Sigma), which is a HIF-1α inhibitor and 50 nM of QNZ (S4902; Selleckchem), which is an NFkB inhibitor, were added to cells cultured in control and uremic serum for 24 h. Luciferase activity was measured according to the manufacturer’s protocols. HIF luciferase activity was measure in normoxia (21% O_2_) and hypoxia (1% O_2_) using a hypoxia chamber.

### Statistical analysis

All results were expressed as mean and standard error of the mean for normalized data or median and interquartile range for non-normalized data. Data were analyzed using two-tailed t tests for comparisons between two groups for normalized data and Mann–Whitney U test for non-normal data. A p value of ≤ 0.05 was considered statistically significant.

## Supplementary Information

Supplementary Information.
