# Supplementary material for: Macrophage and adipocyte interaction as a source of inflammation in kidney disease
Source: Sci Rep. 2021 Feb 3;11:2974. doi: 10.1038/s41598-021-82685-4 (PMC7859223; doi:10.1038/s41598-021-82685-4)

Macrophage and adipocyte interaction as a source of inflammation in kidney disease

Cristina Martos-Rus, MS, Goni Katz-Greenberg, MD, MS, Zhao Lin, MD, Eurico Serrano, MS, Diana Whitaker-Menezes, MS, Marina Domingo-Vidal, MS, Megan Roche, BS, Kavitha Ramaswamy, MD, Douglas C. Hooper, PhD, Bonita Falkner, MD, Maria P Martinez Cantarin, MD, MS

SUPPLEMENTARY TABLE OF CONTENTS

Supplementary Table 1 - TaqMan Assays used in RT-qPCR experiments

Supplementary Table 2 - Antibodies used in flow cytometry staining

Supplementary Figure 1 - Representative images from subcutaneous adipose tissue samples stained with CD163 macrophage marker

Supplementary Figure 2 - Adipose tissue macrophage cell counts and nonnuclear fractions in uremic patients versus controls stratified by BMI

Supplementary Figure 3 - Adipose tissue adipocyte size in uremic patients versus controls stratified by BMI.

Supplementary Figure 4 – Uncropped western blots from Figure 1.

Table S1. TaqMan Assays used for RT-qPCR

Gene	Assay ID	Target species
CCL2	Mm00441242_m1	Mouse
IL-1α	Mm00439620_m1	Mouse
IL-6	Mm00446190_m1	Mouse
TNF-α	Mm00443258_m1	Mouse
GAPDH	Mm99999915_g1	Mouse
ABCA1	Hs00194045_m1	Human
ARG1	Hs00163660_m1	Human
CCL2	Hs00234140_m1	Human
CD36	Hs00354519_m1	Human
IL-1α	Hs00174092_m1	Human
IL-6	Hs00174131_m1	Human
IL-10	Hs00961622_m1	Human
NOS2/iNOS	Hs01075529_m1	Human
PLIN2	Hs00605340_m1	Human
TNF-α	Hs00174128_m1	Human
GAPDH	Hs99999905_m1	Human

Table S2. Antibodies used for flow cytometry staining

Antigen	Clone	Fluorochrome	Source	Reference
Mouse CD11b	M1/70	FITC	BioLegend	101206
Mouse CD11c	HL3	BV421	BD Horizon	562782
Mouse CD163/M130	Polyclonal	Cy5	Bioss Antibodies	bs-2527R-Cy5
Mouse CD204	2F8	PE	Invitrogen	MA5-16496
Mouse CD206	C068C2	PE/Cy7	BioLegend	141719
Mouse CD45	104	PerCP-Cy 5.5	BD Pharmingen	561096
Mouse CD64	X54-5/7.1	FITC	BioLegend	139316
Mouse CD11c	N418	PE/Cy7	BioLegend	117317
Human CD45	HI30	PerCP-Cy 5.5	BD Pharmingen	564106
Human CD14	M5E2	FITC	BD Pharmingen	555397
Human CD11c	Bu1	PE/Cy7	BioLegend	337215
Human CD206	19.2	PE-CF594	BD Horizon	564063

Figure S1

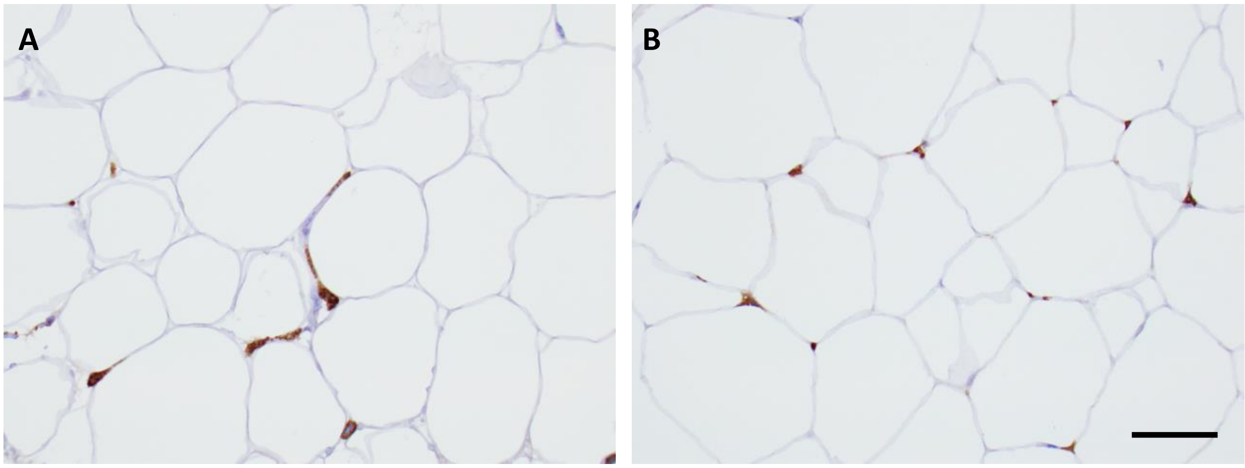

CD163 staining with nuclei

CD163 staining without nuclei
Non-nuclear fractions

Bar = 50 microns

Figure S1. Representative images from subcutaneous adipose tissue samples stained with CD163 macrophage marker.
(**A**) Representative image showing CD163 positivity associated with macrophage nuclei. (**B**) Staining depicting CD163 positivity not associated with nuclei or the non-nuclear fractions. Original mag 40x, bar equals 50 microns.

Figure S2

A **B**

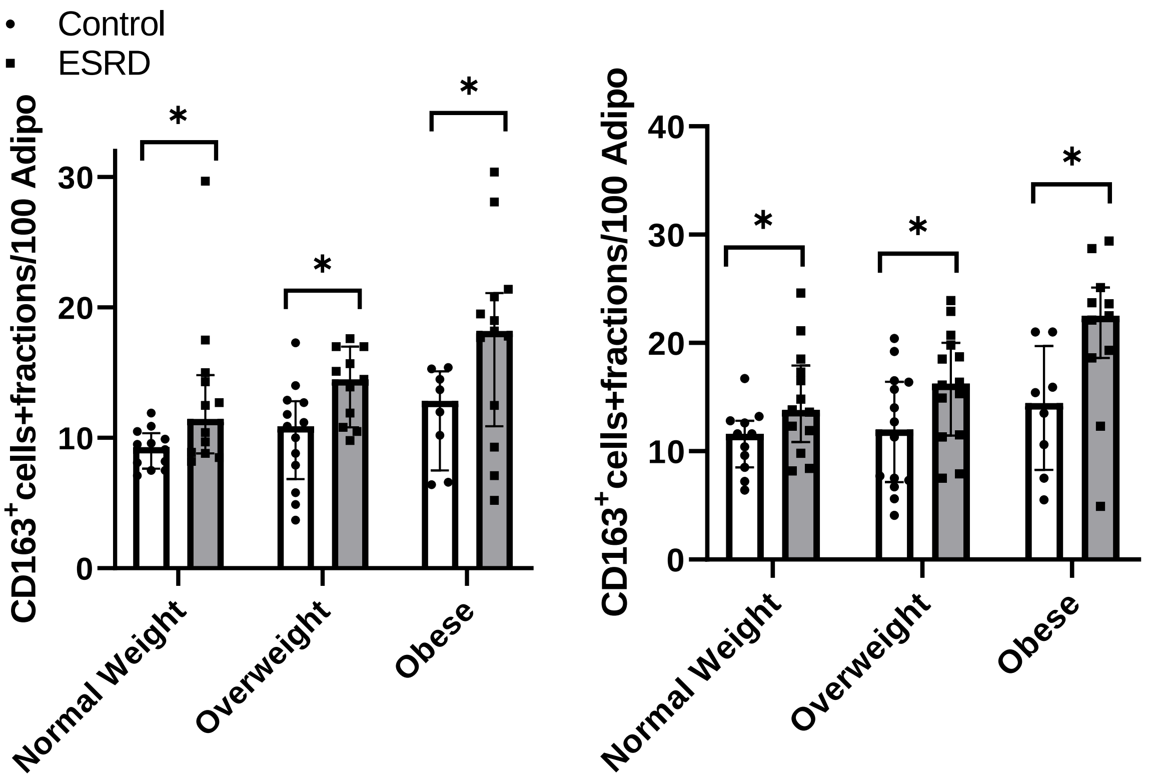

Figure S2: Adipose tissue macrophage cell counts and nonnuclear fractions in uremic patients versus controls stratified by BMI.

(**A**) Number of CD163+ cells and non-nuclear fractions per 100 adipocytes in subcutaneous adipose tissue. (**C**) Number of CD163+ cells and non-nuclear fractions per 100 adipocytes in visceral adipose tissue. *p≤0.05comparing controls and ESRD participants in each adipose tissue depot or each strata of BMI. Mann- Whitney U test, error bars represent IQR. BMI, body mass index. ESRD, end stage renal disease

Figure S3

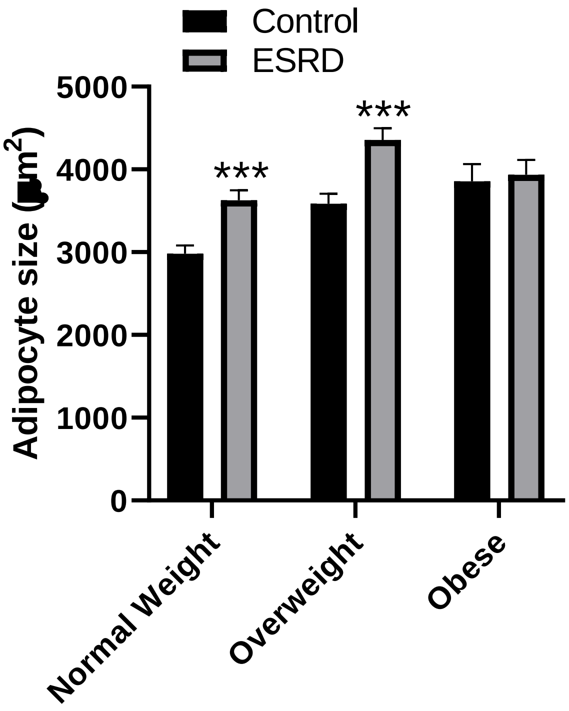

Figure S3: Adipocyte size in visceral adipose tissue of uremic patients versus controls stratified by BMI. ***p<0.001 comparing controls and ESRD participants in each adipose tissue depot or each strata of BMI. Unpaired t test, error bars represent SEM BMI, body mass index. ESRD, end stage renal disease.

Figure S4: Uncropped western blots, form Figure 1. Membrane fractions of 3T3L1 cells exposed to normal and uremic serum

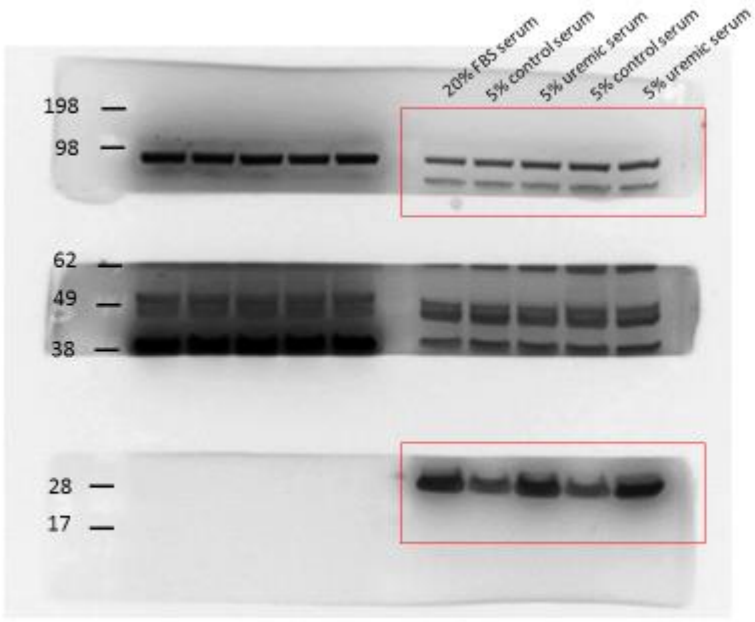

Figure S4: Uncropped western blots from Figure 1. Cytosolic fractions of 3T3L1 cells exposed to normal and uremic serum

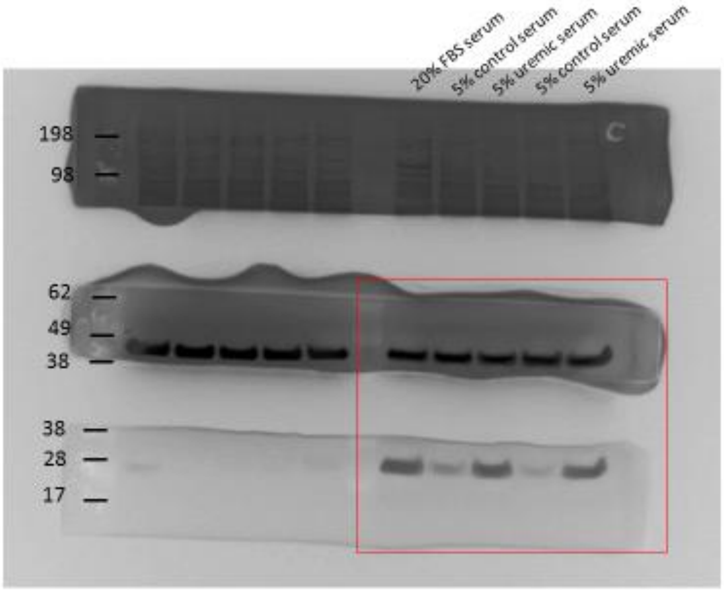

Supplement: Supplementary file 1 — Supplementary Information. [file 41598_2021_82685_MOESM1_ESM.pdf]
